# Supplementary material for: Yiai Fuzheng decoction inhibits triple-negative breast cancer by remodeling the immune microenvironment
Source: Front Immunol. 2025 Sep 30;16:1615631. doi: 10.3389/fimmu.2025.1615631 (PMC12518410; doi:10.3389/fimmu.2025.1615631)
Supplement: Supplementary file 2 [file DataSheet1.zip › Supplementary File 2/Treatment/D_vs_H/07_Pathway_Analysis/Pathway_Analysis/Sig_Pathways_ID/Pyruvate metabolism.pdf]

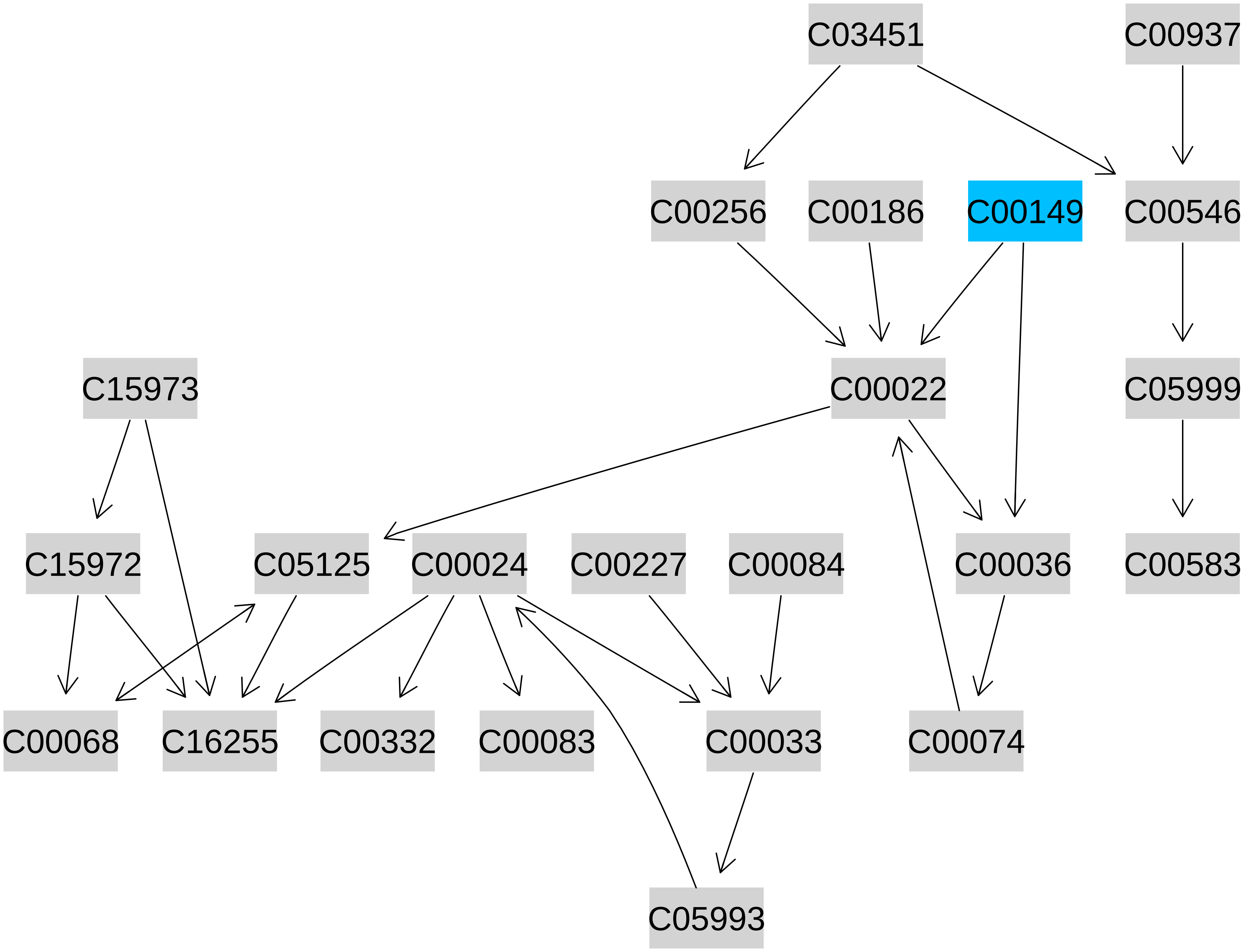

C03451
C00937
C00256
C00186
C00149
C00546
C15973
C00022
C05999
C15972
C05125
C00024
C00227
C00084
C00036
C00583
C00068
C16255
C00332
C00083
C00033
C00074
C05993
